# Evaluating an Intervention Program Using WeChat for Patients With Chronic Obstructive Pulmonary Disease: Randomized Controlled Trial

**DOI:** 10.2196/17089

**Published:** 2020-04-21

**Authors:** Yuyu Jiang, Fenglan Liu, Jianlan Guo, Pingping Sun, Zhongyi Chen, Jinping Li, Liming Cai, Hongqing Zhao, Ping Gao, Zhaosheng Ding, Xiaoliang Wu

**Affiliations:** 1 Research Office of Chronic Disease Management and Rehabilitation Wuxi School of Medicine Jiangnan University Wuxi China; 2 Wuxi School of Medicine Jiangnan University Wuxi China; 3 Affiliated Hospital of Jiangnan University Wuxi China; 4 Wuxi Second People's Hospital Wuxi China; 5 Wuxi Second Hospital of Traditional Chinese Medicine Wuxi China; 6 Rongjun Hospital of Jiangsu Province Wuxi China; 7 Huishan District Rehabilitation Hospital Wuxi China

**Keywords:** chronic obstructive pulmonary disease, randomized controlled trial, self-efficacy, telemedicine, the eHealth enhanced chronic care model, WeChat

## Abstract

**Background:**

The application of telemedicine in home pulmonary rehabilitation interventions for the management of patients with chronic obstructive pulmonary disease (COPD) has achieved promising results.

**Objective:**

This study aimed to develop a WeChat official account (Pulmonary Internet Explorer Rehabilitation [PeR]) based on social media. It further evaluated the effect of PeR on the quality of life, symptoms, and exercise self-efficacy of patients with COPD.

**Methods:**

The functional modules of PeR were developed by a multidisciplinary team according to the electronic health–enhanced chronic care model (eCCM) components. A total of 106 patients were randomly selected (53 in the PeR group and 53 in the outpatient face-to-face group [FtF]). Pulmonary rehabilitation intervention was conducted for 3 months, and the outcome was observed for 3 months. The primary outcome was patient quality of life measured with the COPD assessment test (CAT). The secondary outcomes were evaluated using the modified Medical Research Council scale (mMRC), exercise self-regulatory efficacy scale (Ex-SRES), and St George’s Respiratory Questionnaire (SGRQ).

**Results:**

The intention-to-treat analysis was used in the study. A total of 94 participants completed the 6-month pulmonary rehabilitation program. No statistically significant differences were observed in CAT (*F*_1,3_=7.78, *P*=.001), Ex-SRES (*F*_1,3_=21.91, *P*<.001), and mMRC scores (*F*_1,3_=29.64, *P*<.001) between the two groups with the variation in time tendency. The Ex-SRES score had a significant effect on the CAT score (*P*=.03). The partial regression coefficient of Ex-SRES to CAT was 0.81, and Exp (B) was 2.24.

**Conclusions:**

The telemedicine technology was effective using the eCCM combined with a behavioral intervention strategy centering on self-efficacy. Pulmonary rehabilitation at home through PeR and FtF could improve the sense of self-efficacy and quality of life and alleviate symptoms in patients with COPD.

**Trial Registration:**

Chinese Clinical Trial Registry ChiCTR1900022770; https://tinyurl.com/tmmvpq3

## Introduction

Chronic obstructive pulmonary disease (COPD) is a chronic and progressive respiratory disease [1]. In 2020, COPD is expected to become the third leading cause of death [2] and the fifth leading economic burden of disease [3].

Pulmonary rehabilitation is an important component of COPD treatment and management [4]. “Pulmonary rehabilitation is a comprehensive intervention based on a thorough patient assessment, followed by patient-tailored therapies that include, but are not limited to, exercise training, education, and behavioral change designed to improve the physical and psychological conditions of people with chronic respiratory disease and promote the long-term adherence to health-enhancing behaviors” [5]. Pulmonary rehabilitation could alleviate symptoms, enhance activity tolerance, improve quality of life, and reduce the burden of medical and health service system [6]. Despite the acknowledged benefits, participation and completion of pulmonary rehabilitation training have not lived up to expectations [7]. The standard duration of pulmonary rehabilitation training at home is 8 to 12 weeks. Patients needed long-term maintenance training to ensure the effect of pulmonary rehabilitation [4]. In the United Kingdom, less than 1.5% of patients with COPD receive pulmonary rehabilitation each year [8]. A clinical research showed that only 42% of patients with COPD successfully accomplished pulmonary rehabilitation training [7]. According to the American Thoracic Association and the European Respiratory Association, pulmonary rehabilitation has many limitations, such as insufficient resources for pulmonary rehabilitation, low proportion of medical insurance distribution, and lack of professional health care providers [9]. In addition, other elements (eg, transport, mobility of population, distance, and training location) also make patients with COPD incapable of requesting, participating, and persisting in pulmonary rehabilitation training [10].

Telemedicine service mode can be described as the use of electronic information and communication technology by professional health care providers to provide and support health care to patients in case of long distances [11]. The implementation of pulmonary rehabilitation through remote technology can not only reduce the medical service demand and expenses of patients with COPD but also improve the accessibility of service projects, solve the difficulty of transportation and distance during training, and expand the programs to remote areas [12]. Moreover, in response to the low adherence rate of patient pulmonary rehabilitation training, researchers intended to modify the treatment of chronic respiratory diseases by developing behavioral change interventions [13]. The systematic review by McCullough et al [13] included 46 studies; 19 of them applied 12 different behavioral change theories, among which self-efficacy theory and social cognition theory were used in several studies. Notably, few studies combined behavioral intervention strategies with remote technologies in COPD. Lorig et al [14] implemented internet-based chronic disease self-management projects in 958 patients with chronic diseases that included password protection, interactive network teaching, and health education. The content of health education included personalized sports design, cognitive symptom management, negative emotion management, drug overview, physician-patient communication, and healthy diet. The results showed that providing self-management support through the internet could effectively improve the health status of patients, and it was a feasible choice to replace face-to-face self-management [14].

The advantage of interventions based on theoretical models has been confirmed by researchers [15]. Theoretical models can help researchers conduct clinical research better by observing the relationship between telemedicine content, behavioral mechanisms, and expected outcomes from a holistic perspective [16]. The electronic health–enhanced chronic care model (eCCM) is a theoretical model of telemedicine intervention proposed by Gee and colleagues [17] based on the CCM (chronic care model). The CCM is currently considered the best comprehensive evidence for chronic disease prevention and management interventions [18]. The eCCM is the innovation of CCM and electronic health (eHealth) [17]. No transformation study about the eCCM or other theory based on the eCCM has been published to date.

This study aimed to construct functional modules of Pulmonary Internet Explorer Rehabilitation (PeR, mobile technology, a free social media WeChat official account) according to the features of eCCM components. Meanwhile, behavioral intervention strategies centered on self-efficacy were included to evaluate the effect of PeR’s application. It was hypothesized that PeR could relieve symptoms in patients with COPD and improve their self-efficacy and quality of life.

## Methods

### Study Design

This study was a 6-month randomized controlled trial in which standardized pulmonary rehabilitation intervention lasted for 3 months, and the rehabilitation observation period was 3 months. The research assistant generated the random number sequence using a random assignment sequence table. Patients were randomly divided into intervention and control groups according to the ratio of 1:1. The intervention group received PeR management on the basis of evaluation, including respiratory training, sports training, diet guidance, medication knowledge, and so forth. Patients reported their training records at least once a week. The control group received the same content as the intervention group during an outpatient face-to-face (FtF) intervention. Patients reported their pulmonary rehabilitation training records by mobile phone. The data of patients were collected by research assistants (those who did not know about the grouping) at baseline and in the third and sixth months. The study passed the ethical review of Wuxi Medical College of Jiangnan University (JNU20190318IRB61) and was registered with the Chinese Clinical Trial registry [ChiCTR1900022770]. The Consolidated Standards of Reporting Trials (CONSORT) checklist is in Multimedia Appendix 1.

### Recruitment Processes

From October 1, 2018, to October 7, 2019, patients were recruited from the communities in Wuxi through leaflets, posters, and face-to-face communication. The pulmonary rehabilitation intervention was carried out. The inclusion criteria were aged 60 years and older, confirmed diagnosis of COPD according to the diagnosis and treatment guidelines for chronic obstructive pulmonary disease [19], forced expiratory volume in 1 second (FEV1)/forced vital capacity (FVC) ratio of <0.7, FEV1<80% predicted, and use of WeChat for effective communication. The exclusion criteria included patients with mental disorders, cognitive disorders, and limb dysfunction; patients with unstable heart disease or arrhythmia requiring drug intervention; patients with a history of myocardial infarction or cerebral infarction in the previous year; patients too weak to perform the muscle strength test; patients with hypertension that could not be controlled with drugs; and patients with a history of syncope after exercise. Patients who met the criteria signed the informed consent form. The baseline data measurement was organized by community doctors, nurses, and research assistants, including sex, age, education level, disease severity, and other demographic and sociological information. The St George’s Respiratory Questionnaire (SGRQ), COPD assessment test (CAT), the modified Medical Research Council scale (mMRC), and exercise self-regulatory efficacy scale (Ex-SRES) assessments were completed.

### Development of the Pulmonary Rehabilitation Intervention Program

Before the intervention, respiratory experts, clinicians, rehabilitation practitioners, nurses, research assistants, software engineers, user interface designers, and patients with COPD formed a multidisciplinary team. The team discussed and developed the pulmonary rehabilitation intervention program. The core part of the intervention program included respiratory training, diet guidance, medication knowledge, and exercise training. The implementation of the intervention program was divided into two types: PeR and outpatient FtF intervention. Respiratory training included lip contraction breathing and abdominal breathing. Diet guidance was to make a diet plan according to the standard weight, physical labor intensity, intake proportion of three major nutrients (proteins, carbohydrates, and lipids), and diet preference of patients. Medication knowledge was health education of routine drug use. Exercise training included upper extremity training, lower extremity training, and balance training. The intensity of exercise training was determined and adjusted according to patients’ target heart rate and the score of conscious exertion. The target heart rate was calculated using the Karvonen formula, conscious exertion was calculated using level 13 on the rate of perceived exertion, and patients’ slight fatigue was the best exertion. Training frequency was no less than 3 times a week, and training time was 20 to 30 minutes each time. Patients chose their own time of home-based pulmonary rehabilitation training. Within 3 months of pulmonary rehabilitation intervention, patients in the PeR group reported pulmonary rehabilitation training at least once a week through PeR, and patients in the traditional FtF group reported once a week by telephone. The program was adjusted according to patient reports of home-based pulmonary rehabilitation training. Patients in both groups were not required to report their condition for 3 months during the rehabilitation observation period.

### Development of the Pulmonary Internet Explorer Rehabilitation App

**Figure 1:**
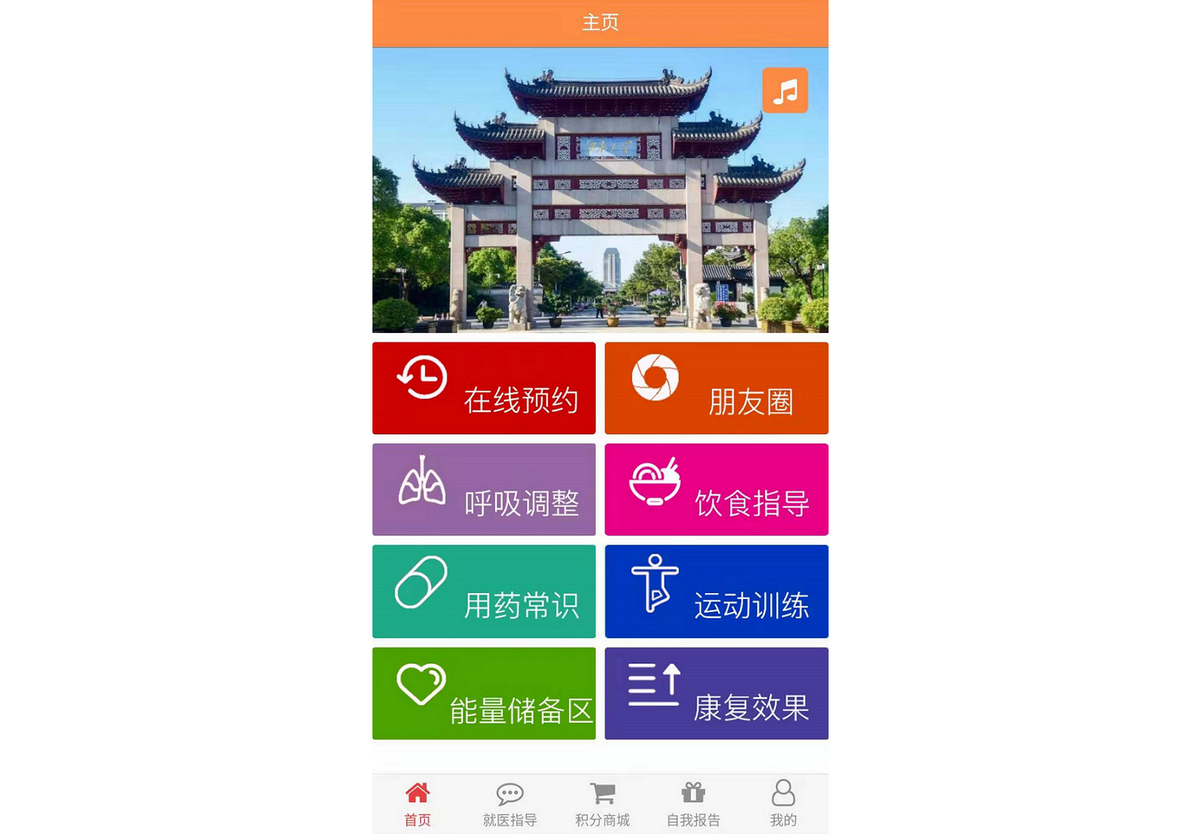
Functional modules of Pulmonary Internet Explorer Rehabilitation on the WeChat terminal.

The focus group method was applied in this study. The multidisciplinary team developed the functional modules of PeR according to eCCM components and the corresponding characteristics. The mapping table of PeR function module is shown in Table 1. PeR is a WeChat official account for patients with stable COPD. It uses the internet, mobile phones, computers, and WeChat to achieve community pulmonary rehabilitation for patients with COPD. The PeR includes two ports: the computer end and the WeChat end. The computer end is operated and managed by health professionals. Patients with COPD can see the functional modules of PeR on the WeChat terminal (Figure 1). The purpose of developing PeR was to allow more patients with COPD to receive pulmonary rehabilitation using free social media and help primary health institutions lacking rehabilitation resources realize the economic intervention and management of COPD pulmonary rehabilitation with the help of WeChat. PeR protects the privacy and data security of patients through access control and permission control, patient data transmission and anonymity, redundant storage, and data backup. PeR has obtained the computer software copyright registration certificate (Multimedia Appendix 2), and the intellectual property is protected by the State Copyright Administration of the People’s Republic of China. A screenshot of the English version of the app is shown in Multimedia Appendix 3.

**Table 1:** Mapping table of the Pulmonary Internet Explorer Rehabilitation function module.

Components of the eCCM^a^	Characteristic	PeR^b^ function module
eCommunity	Participation, active, self-management	PeR moments
Communication and the addition of the complete feedback loop	Communication, complete feedback Control over the timing	Appointment for medical treatment Medical guidance
Clinical information systems enhancements	Register Database Support access	Register log-in Rehabilitation effect database Self-report database
Self-management support enhancements	Education (information) Behavior support (an aid to behavioral change)	Respiratory training Diet guidance Medication knowledge Exercise training Integral mall
Add self-efficacy resource component	Mastery experience Alternative experience Verbal persuasion Physiological and emotional state	Respiratory training Diet guidance Medication knowledge Exercise training Integral mall

^a^eCCM: electronic health–enhanced chronic care model.

^b^PeR: Pulmonary Internet Explorer Rehabilitation.

### Pulmonary Internet Explorer Rehabilitation App Moments

The implementation process on WeChat Moments was designed according to the four resources of self-efficacy theory (mastery experience, verbal persuasion, physiological and emotional state, and alternative experience). In PeR Moments, patients with COPD can upload their rehabilitation training pictures or speeches. Other patients and health professionals can interact with them by commenting or giving thumbs up, thus promoting not only peer support between patients but also communication between doctors and patients. Moreover, an incentive system was designed to encourage patients with COPD in their participation. To illustrate, patients obtained scores by posting PeR Moments, after which they could exchange prizes by accumulating scores to a certain amount. Meanwhile, the standardized pulmonary rehabilitation training patients shared with the PeR Moment was set as an example to encourage remaining patients to persist in training.

### Appointment for Medical Treatment and Medical Guidance

Patient baseline situations were measured by health care personnel in community health centers, while professionals (medical personnel in the multidisciplinary team) developed the pulmonary rehabilitation program. After that, pulmonary rehabilitation training of patients with COPD was initiated and managed by professionals through PeR. Patients could get the electronic pulmonary rehabilitation prescription in the medical guidance module and contact nursing members online in the same module if the training program needed to be adjusted. When the disease worsened, patients could communicate with nursing members after making an appointment. Nursing members recommended appropriate professionals for further treatment according to their condition.

### Register Log-in, Rehabilitation Effect Database, and Self-Report Database

PeR included a registration center, rehabilitation effect database, and self-report database. The rehabilitation effect database was uploaded by a research assistant through a computer at baseline and after 3 and 6 months of testing. The self-report database was completed and uploaded by patients themselves on the WeChat end. Two community nurses were assigned by the community hospital to manage all patient data. Patients could browse and obtain all personal data in the database on the WeChat end.

### Respiratory Training, Diet Guidance, Medication Knowledge, Exercise Training, and Integral Mall

The audio and graphic versions of the modules on respiratory training, diet guidance, medication knowledge, and exercise training function were designed by a multidisciplinary team. Patients could choose different forms according to their own preferences. An integral mall was designed to encourage patients to generate network behavior so as to strengthen the function of the PeR Moments. Supervision of rehabilitation training behavior was increased to promote the maintenance of rehabilitation training behavior.

### Pulmonary Rehabilitation Intervention

#### Intervention Process of the Pulmonary Rehabilitation Group

**Figure 2:**
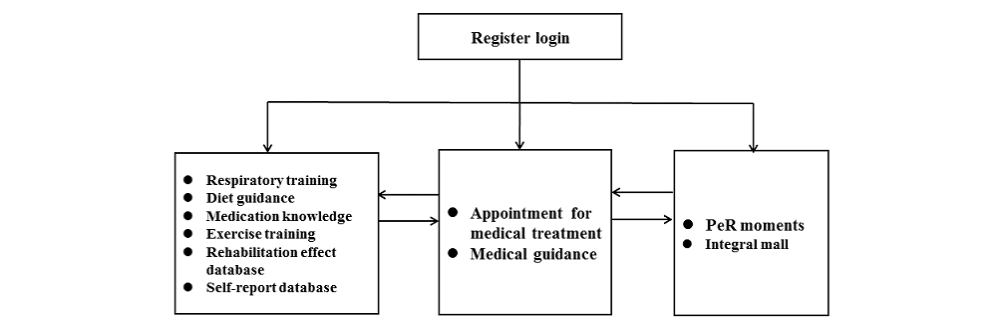
Patient log-in process.

Before the start of pulmonary rehabilitation intervention, patients first received face-to-face training from medical and health care professionals. The training contents included how to obtain, understand, and apply the intervention program of pulmonary rehabilitation through PeR; how to complete self-report through PeR; how to share their own pulmonary rehabilitation training; and how to communicate and interact with peer patients and health care professionals through PeR. At the same time, patients received online training manuals. At the beginning, patients registered and logged on to PeR. Health care professionals evaluated patients, entered electronic pulmonary rehabilitation prescriptions, and distributed training aids such as resistance bands. Patients could view their electronic pulmonary rehabilitation prescription in the medical guidance module and the evaluation report in the rehabilitation effect database. Patients completed the home-based pulmonary rehabilitation training, completed the self-assessment report, and uploaded their training records in Moments. Health care professionals guided patients by reviewing the uploaded content. In the case of acute exacerbation of a patient’s condition during this period, one could get in touch with health care professionals through the module of guidance and appointment for medical treatment, who would arrange the medical treatment schedule immediately. During the whole process of pulmonary rehabilitation intervention, patients could earn gifts in the integral mall using their own points and receive them in the third and sixth months of retest evaluation. The patient log-in process in the interface is shown in Figure 2.

#### Intervention Process of the Face-to-Face Group in the Outpatient Department

Without using WeChat, the FtF group in the outpatient department received the same intervention as the PeR group, with the same pulmonary rehabilitation training equipment. According to the different training objectives, patients in the intervention and control groups all obtained one elastic band, and the elastic grade of the elastic band was consistent with the training objectives.

### Follow-Up Process

The primary and secondary evaluation indexes were collected at baseline and in the third and sixth months. Patients in the intervention and control groups were tested separately face to face in the community hospital. Three days before the retest of each stage, the nurses in the community hospital informed patients to participate in the retest. Patients in the intervention group received an appointment reminder through PeR, and patients in the control group were informed by mobile phone. Patients reserved the right to withdraw from the study at any time during the study period.

### Demographic and Sociological Information

The demographic and sociological information of patients in the intervention and control groups, including age, sex, education level, disease severity, and body mass index (BMI), were collected. A baseline comparison was then made.

### Main Evaluation

CAT is a simple and easy-to-use health assessment tool for clinical practice to help patients and clinicians evaluate the symptoms and effects of diseases in a quantitative manner and to promote communication between patients and doctors. It includes 8 items in total. Each item evaluates a state, such as cough, expectoration, chest distress, energy, and so forth. Based on the state described by each item, the score range is from the best (score 0) to the worst (score 5). The total CAT score is the sum of the scores of all items. The higher the total score, the worse the patient’s health [20].

### Secondary Evaluation

SGRQ can be used as an important tool to evaluate the symptoms, pulmonary function, general well-being, and quality of life of patients with COPD and the effectiveness of medical services [21-23]. The scale contains 50 items, which are divided into 3 dimensions: symptom, activity, and influence. The score of the scale is from 0 to 100, which is calculated by a certain weight between the three subscales and the total scale. The higher the score, the worse the health status of patients with COPD. A score of 0 indicates that the disease caused no damage to a patient, and a score of 100 indicates maximum damage [20].

Ex-SRES was developed based on motor disorders to evaluate exercise self-efficacy [24]. Ex-SRES has 16 items reflecting patient confidence to continue to exercise under the conditions of bad weather, pain, exercise alone, busy, no support from others, lack of oxygen, vacation, fatigue, and no desire to exercise. Each item is measured with 1 to 10 points; 1 point means no self-confidence and 10 points mean very high self-confidence. The higher the score, the higher the confidence to keep exercising [24]. Ex-SRES is a single-factor structure with good internal consistency; the Cronbach coefficient is 0.917 [24]. Studies showed that Ex-SRES positively influenced adherence to regular exercise in patients with COPD [25].

mMRC is a questionnaire with 5 dimensions that provides a measurement method for perceived dyspnea. It is divided into 0 to 4 points: 0 points means do not feel dyspnea generally except for strenuous exercise; 1 point means short of breath when fast-walking on flat ground or when going uphill; 2 points means walking on flat ground slower than peers due to shortness of breath or having to stop walking to catch one’s breath; 3 points means shortness of breath when walking on level ground for 100 meters or several minutes; and 4 points means inability to leave the room due to shortness of breath. The higher the score of the scale, the more severe the dyspnea [26].

### Sample Size

The minimal clinically important difference in CAT score was 0.5 points. Previous studies showed that it could be improved by 2 points [19], and the estimated standard deviation was 4 points. With the test level of 0.05 on both sides and 80% test efficacy, the sample size estimation method was used for each group’s measurement data. When the sample number of each group was equal, at least 31 cases were needed in each group. According to the estimation of 15% dropout rate in the study, at least 38 cases were needed in each group, and at least 76 cases were included in the sample size. The final sample size was 106.

### Data Analysis

The intention-to-treat analysis was used in the study. The missing values of lost subjects used the last observation carried forward for analyzing. SPSS Statistics version 20.0 (IBM Corp) was used for data analysis, with the statistical significance set at *P*<.05. Sex, course of disease, education level, smoking status, and disease classification were analyzed using chi square tests. Age and BMI were analyzed by independent samples *t* tests. The changes in CAT, Ex-SRES, and mMRC scores in different intervention time periods (0, 3, and 6 months) were studied using the repeated measures analyses of variance. The paired samples *t* test was used to analyze the SGRQ at baseline and 6 months after the intervention. The effect comparison between the two groups in the sixth month used the difference between before and after the intervention for independent samples *t* test analysis. The intervention methods, intervention time, self-efficacy, and interaction among the aforementioned factors were analyzed using the regression modeling of the generalized estimating equation (GEE) of autocorrelation work structure to explore the significance of these factors in improving the quality of life of patients.

## Results

### Process of Recruitment and Research

**Figure 3:**
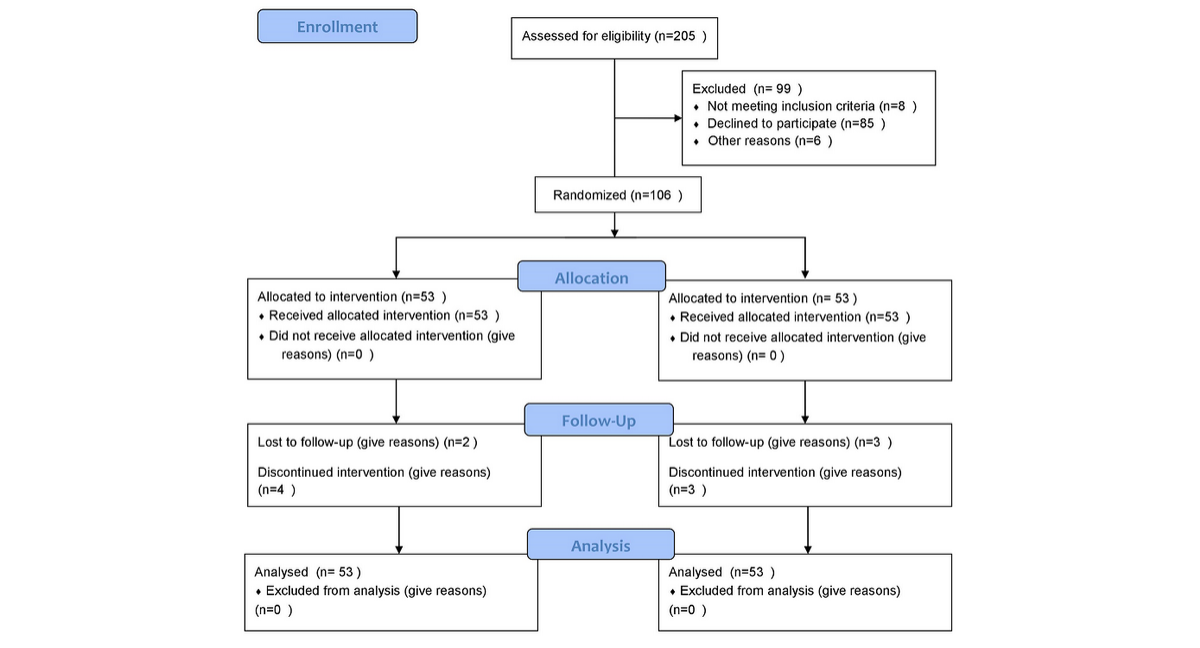
Consolidated Standards of Reporting Trials flowchart.

The recruitment of patients with COPD started on October 1, 2018, and the process of follow-up was completed by October 7, 2019. Figure 3 shows the CONSORT flowchart of this study, which describes in detail the process of recruiting patients, implementation of structured intervention program, observation effect, and loss to follow-up. During the study, 12 patients dropped out, and the dropout rate was 11.3%. In the PeR group, 6 patients dropped out, including 3 patients who felt that rehabilitation was of no use and dropped out immediately, 1 patient who got worse, and 2 who moved to other places. In the FtF group, 6 patients dropped out, including 2 patients who moved to other places, 2 patients who got worse, and 2 patients who felt rehabilitation was of no use.

### Basic Demographic Characteristics and Measurements

No statistically significant difference was observed in socioeconomic information and measurement at baseline (*P*>.05; Table 2).

### Main Evaluation Indexes

**Figure 4:**
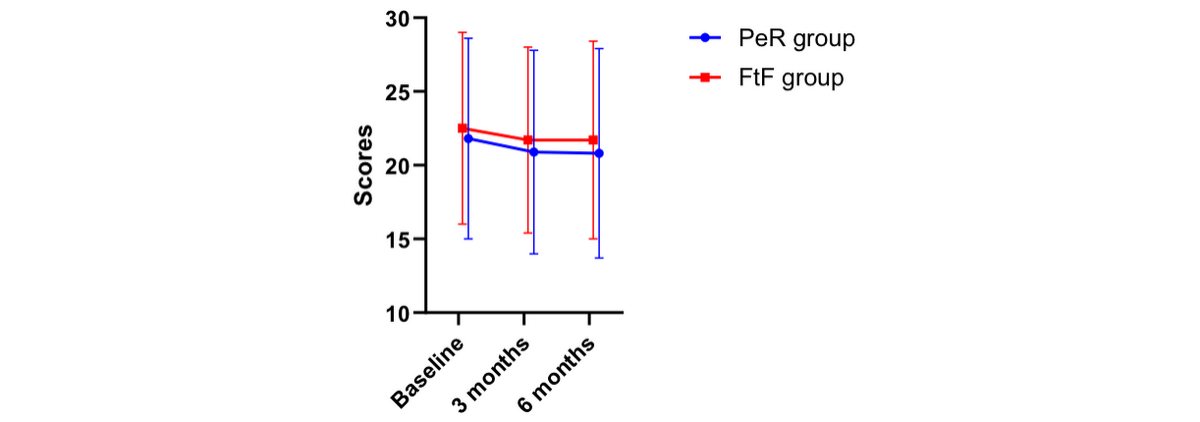
Chronic obstructive pulmonary disease assessment test score and its change over time.

CAT was used to evaluate the health status of patients. A tendency to change in the CAT score was observed within 6 months (*P*=.001). The change trend of the CAT score over time is shown in Figure 4. No difference was found between the two groups (*P*=.53), and no interaction was observed between time and groups (*P*=.98). The results are shown in Table 3. The CAT score in the two groups changed from baseline to the third month, from the third month to the sixth month, and from baseline to the sixth month (*P*=.002, *P*=.70, and *P*=.001, respectively; Table 4).

**Table 2:** Basic demographic characteristics and measurements of the two groups.

Characteristics		PeR^a^ group, (n=53)	FtF^b^ group, (n=53)	Total, (n=106)	*χ*^2^(δφ)	*t* (df)	*P* value
**Sex, n (%)**		—	—	—	0.06 (1)	—	.50
	Male	44 (83)	43 (81)	87 (82)	—	—	—
	Female	9 (17)	10 (19)	19 (18)	—	—	—
Age, mean (SD)		70.92 (6.38)	71.83 (7.60)	—	—	–0.67 (104)	.51
BMI^c^, mean (SD)		22.21 (3.52)	21.27 (2.36)	—	—	1.62 (90.84)	.11
**Disease duration, n (%)**		—	—	—	0.05 (1)	—	>.99
	<10 years	16 (30)	15 (28)	31 (29)	—	—	—
	≥10 years	37 (70)	38 (72)	75 (71)	—	—	—
**Education status, n (%)**		—	—	—	3.58 (3)	—	.31
	Primary school	12 (23)	13 (25)	25 (24)	—	—	—
	Middle school	15 (28)	22 (42)	37 (35)	—	—	—
	High school	17 (32)	14 (26)	31 (29)	—	—	—
	Higher school	9 (17)	4 (8)	13 (12)	—	—	—
**Smoking status, n (%)**		—	—	—	0.00 (2)	—	>.99
	No	12 (23)	12 (23)	24 (23)	—	—	—
	Exsmoker	35 (66)	35 (66)	70 (66)	—	—	—
	Current smoker	6 (11)	6 (11)	12 (11)	—	—	—
**Disease classification^d^**, n (%)		—	—	—	6.69 (3)	—	.08
	GOLD II	27 (50.9)	20 (38)	47 (44)	—	—	—
	GOLD III	15 (28.3)	27 (51)	42 (40)	—	—	—
	GOLD IV	11 (21)	6 (11)	17 (16)	—	—	—
**Measurements, mean (SD)**		—	—	—	—	—	—
	CAT^e^	21.79 (6.85)	22.55 (6.48)	—	—	–0.58 (104)	.56
	Ex-SRES^f^	72.25 (38.38)	71.48 (40.76)	—	—	0.10 (104)	.92
	mMRC^g^	2.79 (0.66)	2.75 (0.71)	—	—	0.28 (104)	.78
	SGRQ-S^h^	53.02 (19.90)	51.12 (18.63)	—	—	0.51 (104)	.61
	SGRQ-A^i^	56.44 (23.96)	56.87 (22.47)	—	—	–0.09 (104)	.93
	SGRQ-I^j^	45.82 (24.27)	44.92 (18.69)	—	—	0.22 (104)	.83
	SGRQ-T^k^	50.24 (20.95)	49.57 (17.52)	—	—	0.18 (104)	.86

^a^PeR: Pulmonary Internet Explorer Rehabilitation.

^b^FtF: face to face.

^c^BMI: body mass index.

^d^GOLD: Global Initiative for Chronic Obstructive Lung Disease; GOLD II: FEV1/FVC<70%, 50%≤FEV1<80%; GOLD III: FEV1/FVC<70%, 30%≤FEV1<50%; GOLD IV: FEV1/FVC<70%, FEV1<30%.

^e^CAT: chronic obstructive pulmonary disease assessment test.

^f^Ex-SRES: exercise self-regulatory efficacy scale.

^g^mMRC: modified Medical Research Council scale.

^h^SGRQ-S: St George’s Respiratory Questionnaire–System.

^i^SGRQ-A: St George’s Respiratory Questionnaire–Activity.

^j^SGRQ-I: St George’s Respiratory Questionnaire–Influence.

^k^SGRQ-T: St George’s Respiratory Questionnaire–Total.

**Table 3:** Variation tendency of chronic obstructive pulmonary disease assessment test, exercise self-regulatory efficacy scale, and modified Medical Research Council scale scores in the two groups.

Item and group	CAT^a^		Ex-SRES^b^		mMRC^c^	
	*F*_1,3_	*P* value	*F*_1,3_	*P* value	*F*_1,3_	*P* value
Time	7.78	.001	21.91	<.001	29.64	<.001
Group	0.39	.53	0.23	.63	0.00	>.99
Time×group	0.02	.98	2.02	.14	0.8	.43

^a^CAT: chronic obstructive pulmonary disease assessment test.

^b^Ex-SRES: exercise self-regulatory efficacy scale.

^c^mMRC: modified Medical Research Council scale.

**Table 4:** Comparison of chronic obstructive pulmonary disease assessment test, exercise self-regulatory efficacy scale, modified Medical Research Council scale, and St George’s Respiratory Questionnaire scores between the two groups before and after intervention.

Outcomes		Baseline	After intervention (3 months)	After intervention (6 months)	*F*_1,3_^a^/*t*_3_^b^ (*P* value)
**CAT^c^**					
	PeR^d^ group, mean (SD)	21.79 (6.85)	20.98 (6.99)	20.85 (7.11)	3.70 (.02)
	FtF^e^ group, mean (SD)	22.55 (6.48)	21.75 (6.25)	21.70 (6.69)	4.17 (.02)
	*F*_1_^f^ (*P* value)	10.14 (.002)	—	—	—
	*F*_2_^g^ (*P* value)	—	0.15 (.70)	—	—
	*F*_3_^h^ (*P* value)	—	—	12.80 (.001)	—
**Ex-SRES^i^**					
	PeR group, mean (SD)	72.25 (38.38)	85.36 (33.18)	80.53 (37.72)	17.22 (<.001)
	FtF group, mean (SD)	71.48 (40.76)	78.49 (33.94)	78.25 (35.40)	6.47 (.008)
	*F*_1_ (*P* value)	54.10 (<.001)	—	—	—
	*F*_2_ (*P* value)	—	3.99 (.05)	—	—
	*F*_3_ (*P* value)	—	—	14.12 (<.001)	—
**mMRC^j^**					
	PeR group, mean (SD)	2.79 (0.66)	2.51 (0.72)	2.40 (0.79)	14.73 (<.001)
	FtF group, mean (SD)	2.75 (0.70)	2.58 (0.69)	2.36 (0.71)	15.77 (<.001)
	*F*_1_ (*P* value)	21.54 (<.001)	—	—	—
	*F*_2_ (*P* value)	—	14.06 (<.001)	—	—
	*F*_3_ (*P* value)	—	—	43.97 (<.001)	—
**SGRQ-S^k^**					
	PeR group, mean (SD)	53.02 (19.90)	—	43.59 (23.63)	3.59 (.001)
	FtF group, mean (SD)	51.12 (18.63)	—	45.33 (22.25)	2.89 (.006)
	*t*^l^ value (*P* value)	—	—	–1.10 (.27)	—
**SGRQ-A^m^**					
	PeR group, mean (SD)	56.44 (23.96)	—	48.74 (24.28)	3.01 (.004)
	FtF group, mean (SD)	56.87 (22.47)	—	53.46 (23.06)	2.89 (.006)
	*t* value (*P* value)	—	—	–1.53 (.13)	—
**SGRQ-I^n^**					
	PeR group, mean (SD)	45.83 (24.27)	—	33.27 (22.86)	3.89 (<.001)
	FtF group, mean (SD)	44.92 (18.69)	—	38.63 (21.88)	3.65 (.001)
	*t* value (*P* value)	—	—	–1.71 (.09)	—
**SGRQ-T^o^**					
	PeR group, mean (SD)	50.24 (20.95)	—	39.66 (20.92)	4.06 (<.001)
	FtF group, mean (SD)	49.57 (17.52)	—	44.24 (19.90)	3.84 (<.001)
	*t* value (*P* value)	—	—	–1.78 (.08)	—

^a^*F*_1,3_: repeated measures analysis of variance.

^b^*t*_3:_ 6 months compared with baseline.

^c^CAT: chronic obstructive pulmonary disease assessment test.

^d^PeR: Pulmonary Internet Explorer Rehabilitation.

^e^FtF: face-to-face.

^f^*F*_1_: 3 months compared with baseline.

^g^*F*_2_: 6 months compared with 3 months.

^h^*F*_3_: 6 months compared with baseline.

^i^Ex-SRES: exercise self-regulatory efficacy scale.

^j^mMRC: modified Medical Research Council scale.

^k^SGRQ-S: St George’s Respiratory Questionnaire–System.

^l^*t*: comparison among groups.

^m^SGRQ-A: St George’s Respiratory Questionnaire–Activity.

^n^SGRQ-I: St George’s Respiratory Questionnaire–Influence.

^o^SGRQ-T: St George’s Respiratory Questionnaire–Total.

### Secondary Evaluation Indexes

SGRQ was used to evaluate the symptom, activity, and influence of patients. Table 4 shows that the SGRQ-S, SGRQ-A, SGRQ-I, and SGRQ-T scores in the PeR group were improved from baseline to the sixth month (*P*=.001, *P*=.004, *P*<.001, and *P*<.001, respectively). The SGRQ-S, SGRQ-A, SGRQ-I, and SGRQ-T scores in the FtF group were improved (*P*=.006, *P*=.006, *P*=.001, and *P*<.001, respectively). In the sixth month, no significant difference was found between the measured value and the baseline value of SGRQ-S, SGRQ-A, SGRQ-I, and SGRQ-T in the two groups (*P*=.27, *P*=.13, *P*=.09, and *P*=.08, respectively).

**Figure 5:**
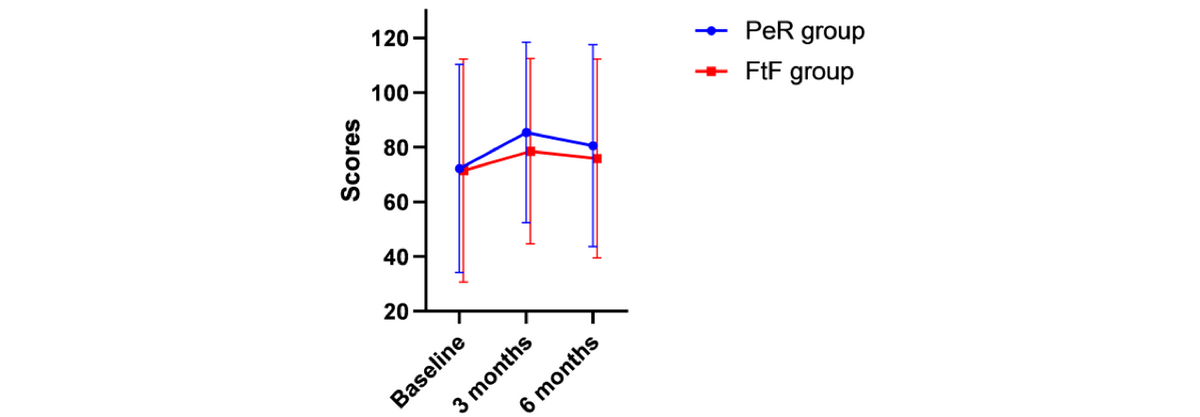
Exercise self-regulatory efficacy scale score and its change over time.

Ex-SRES was used to evaluate the patient sense of self-efficacy. A tendency to change in the CAT score was noted within 6 months (*P*<.001). The change trend of the Ex-SRES score over time is shown in Figure 5. No difference was found between the two groups (*P*=.63), and no interaction was observed between time and groups (*P*=.14). The results are shown in Table 3. The Ex-SRES score in the two groups changed from baseline to the third month, from the third month to the sixth month, and from baseline to the sixth month (*P*<.001, *P*=.05, and *P*<.001, respectively; Table 4).

**Figure 6:**
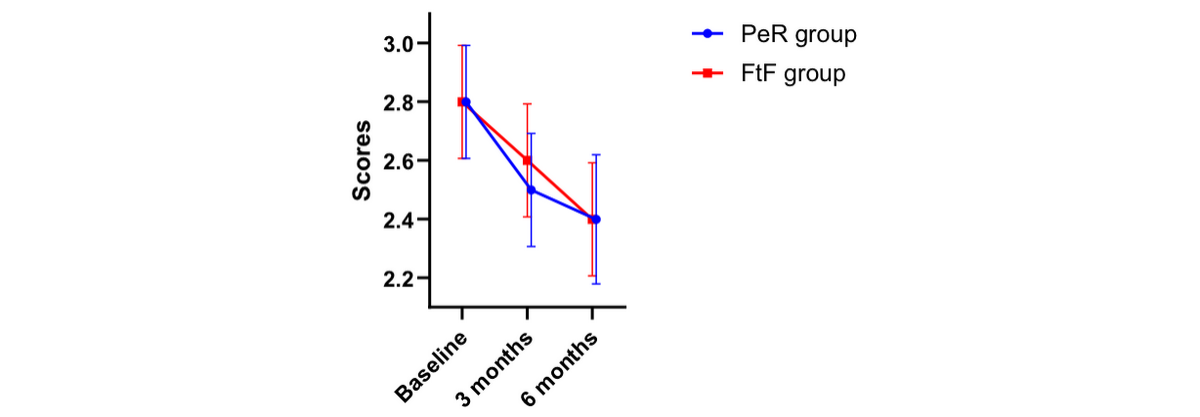
Modified Medical Research Council scale score and its change over time.

mMRC was used to evaluate the status of dyspnea. A tendency to change in the CAT score was observed within 6 months (*P*<.001). The change trend of the mMRC score over time is shown in Figure 6. No difference was found between the two groups (*P*>.99), and no interaction was noted between time and groups (*P*=.43). The results are shown in Table 3. The mMRC score in the two groups changed from baseline to the third month, from the third month to the sixth month, and from baseline to the sixth month (*P*<.001, *P*<.001, and *P*<.001, respectively; Table 4).

### Factors of Methods, Time, and Exercise Self-Regulatory Efficacy Scale Effects on Primary Outcome

Table 5 shows the analysis results using GEE. Only the Ex-SRES had a significant effect on the CAT score (*P*=.03), but the intervention method and time had no significant effect on the CAT score (*P*=.48 and *P*=.84, respectively). The partial regression coefficient of Ex-SRES to CAT was 0.81, and Exp (B) was 2.24. Interaction between intervention methods and Ex-SRES; interaction between intervention methods and intervention time; interaction between Ex-SRES and intervention time; and interaction among intervention methods, Ex-SRES, and intervention time had no statistically significant effect on the CAT score (*P*=.13, *P*=.85, *P*=.09, and *P*=.08, respectively).

**Table 5:** Analysis of the influence of intervention methods, intervention time, and self-efficacy on patients’ quality of life using the generalized estimating equation.

Parameter	*B*	SE	Wald	*P* value	Exp *(B)*
Constant	–1.62	0.33	24.33	<.001	0.20
Intervention methods	–0.25	0.35	0.49	.48	0.78
Ex-SRES^a^	0.81	0.38	4.63	.03	2.24
Intervention time	–0.07	0.35	0.04	.84	0.93
Intervention methods×Ex-SRES	0.75	0.49	2.34	.13	2.12
Intervention methods×intervention time	0.10	0.51	0.03	.85	1.10
Ex-SRES×intervention time	0.97	0.56	2.96	.09	2.64
Intervention methods×Ex-SRES×intervention time	–1.86	1.05	3.16	.08	0.16

^a^Ex-SRES: exercise self-regulatory efficacy scale.

## Discussion

### Principal Findings

It is feasible and effective to implement PeR pulmonary rehabilitation based on the eCCM and self-efficacy theory to help patients with COPD relieve dyspnea symptoms, improve their self-efficacy, and improve their quality of life. The effect was the same as that of the face-to-face intervention.

Three months after the intervention, patient improved CAT scores maintained a subsequent effect and the mMRC scores continued to improve. Meanwhile, the Ex-SRES scores decreased after the peak in the third month instead of a subsequent effect, but it was still an improvement compared with before the intervention. All dimensions and total SGRQ scores of patients improved at the end of the intervention.

Patient Ex-SRES scores correlated with CAT scores. Moreover, the improvement in health was 2.24 times higher in patients with high Ex-SRES scores than in those with low Ex-SRES scores.

Remote technology can be used for information support, information storage and management, telemonitoring, and teleconsultation. Common mobile health technologies include personal computers, tablets, smartphones, and so forth [27,28]. According to the review by McCabe et al [29], the self-management intervention of patients with COPD using mobile technology could improve the quality of life and activity level of patients, and the effect could be maintained for a period of time. However, research by Vorrink et al [30] showed that the evaluation and telemonitoring of physical activity, functional exercise ability, health-related quality of life, and other indicators of patients with COPD using smartphones and websites could not improve the self-efficacy of patients. Thus, it is evident that telemonitoring alone cannot improve the health status of patients.

For patients with COPD, the increase in exercise ability and the change in adaptive behavior are the premises to improve patient health conditions [31]. In a previous study, patients were taught the skills of pulmonary rehabilitation training face to face, and health education content was provided to them. In addition, their training behaviors were evaluated, persuaded, and strengthened using short messaging service. The results showed that patients’ self-efficacy, dyspnea symptoms, and quality of life were all improved [32]. Therefore, for patients with COPD, sports training should be considered as the core of pulmonary rehabilitation program and behavior intervention.

In this study, the incentive function of the integral mall was a way to promote behavioral change and maintenance. In addition, gamification is a good principle. The study by Tabak et al [33] showed that, through telemedicine, gamification strategies could increase patients’ motivation for behavioral change and promote their active lives. This study applied the WeChat official account to carry out pulmonary rehabilitation intervention along with management and provided a pulmonary rehabilitation program centered on sports training for patients with COPD at home. During the process of implementation, the behavioral intervention aimed at enhancing self-efficacy could be realized through the design of WeChat’s functional structure and the application of remote technology.

Many studies suggested that self-efficacy was a key cognitive (motivational) factor in adopting and maintaining self-management behaviors [34,35]. Zarski et al [36] proposed that the systematic development of planning skills and the maintenance of self-efficacy before or during internet-based interventions would help participants successfully complete treatments. Schwarzer’s health action process approach points out that self-efficacy plays an important role in improving and maintaining health behavior [37]. Self-efficacy is a predictor of health and quality-of-life improvement in patients undergoing pulmonary rehabilitation [38].

This study was based on four kinds of information sources proposed by Bandura, mastery experience, alternative experience, verbal persuasion, and good physiological and emotional state, to promote the establishment of patient self-efficacy beliefs. Through the function modules of respiratory training, diet guidance, medication guidance, and sports training, patients could master the knowledge and skills of pulmonary rehabilitation, which could be defined as an information source related to mastery experience. Peer support and doctor-patient communication were promoted through comments and thumbs up interactions in Moments, which not only strengthened the frequency of information source stimulation of verbal persuasion but also built a good virtual social support system for patients’ home-based rehabilitation training. The presentation of successful cases in Moments could construct an alternative experience. On account of the positive incentive function in the integral mall, patient confidence in completing the training could be strengthened and a good mood could be maintained. During the construction of the environment in the aforementioned individual action, social and psychological support environment realized a constantly strengthening behavior intervention process. Further, with the convenience of a mobile terminal across time and space, patients with COPD could continue to receive the stimulation of information sources in their daily life and gradually cultivate the confidence of persisting in long-time sports training. This study combined the strategy of behavior intervention in sociology with the content of telemedicine intervention to provide substantive medical services, making it a new telemedicine service model with warmth and humanistic care. Besides, such research production could provide inspiration for the application of remote technology combined with cognitive behavioral intervention strategies in COPD.

The systematic review by Cruz et al [39] showed that patient perceived difficulties was the reason for the failure to adhere to pulmonary rehabilitation during telemedicine intervention. Thus, organizing more training courses would help patients accept and use remote intervention technology. In this study, preintervention training was used to solve the problem—that is to say, patients were not proficient in technical operation skills. In addition, appointments and medical guidance could integrate medical resources. Patients who were far away from each other could access PeR through free social media and then join the pulmonary rehabilitation program. This was consistent with the concept of making pulmonary rehabilitation an available and affordable project proposed by the World Health Organization [40]. Thanks to the network advantage and easy operation of PeR, the response time of health care professionals was shortened, which helped promote complete feedback between professionals and patients.

The development of internet and mobile devices has promoted the development of telemedicine. Most of the telemedicine technologies applied in countries with developed medical resources intended to telemonitor and manage patients with COPD are expensive, such as remote monitoring systems [39], robots [41], and electronic health systems [42]. Remote technology applied in this study was a WeChat official account developed with certain functions. Due to a large number of patients with COPD in China, some technical and financial limitations still exist in realizing intelligent pulmonary rehabilitation management based on the internet. The number of active users of WeChat in China has exceeded 697 million [43]. WeChat is a widely used and free app on mobile terminals, and its function is similar to that of Facebook [43]. The WeChat app has a positive impact on health behavior. By acquiring information provided on WeChat, health knowledge can be increased, and the ability of public health decision making and action can be improved. The results of a survey indicated that 97.68% of respondents read health information through WeChat [44]. In this study, the network platform based on WeChat was used to implement pulmonary rehabilitation intervention. WeChat is an important information access port for users on mobile terminals. It can realize the interaction and communication between developers and subscribers by pushing graphics, video, and audio through multimedia [45]. At the same time, this study also guaranteed patient privacy and data security by conducting technical strategy, confidentiality agreements, and patient coding. Using free social media to carry out pulmonary rehabilitation intervention and management can provide a reference for similar service projects in countries with resource shortages.

### App Design

The development of telemedicine technology should be a process of interdisciplinary integration. A previous study showed that the continued participation of multiple stakeholders and users was critical to the design and development of successful eHealth solutions [46]. Participants with a multidisciplinary context have different resources and experience, and thus they can innovate and improve the existing service model. At the same time, the user-centered design concept enables the developers of telemedicine technology to give priority to patients’ needs, which helps developers establish specific app functions and hardware as early as possible [47]. The existing methods for user-centered design include workshops and focus groups, paper prototyping, sketching, thinking aloud, scenarios, storytelling, interviews and field studies, questionnaires, and other methods [48]. In this study, respiratory experts, clinicians, rehabilitation practitioners, nurses, research assistants, and patients with COPD all participated in the process of development and application of PeR. Through the establishment of focus groups, the views of different stakeholders were absorbed and intervention content was determined along with the presentation form of PeR (graphic, audio, video, background color, font, font size, etc).

The results of the systematic review showed that the technology acceptance theory and its basic behavior theory could be applied to the acceptance research of telemedicine [49]. At present, technology acceptance models are commonly used. They evaluate the availability and acceptance of telemedicine technology using usefulness cognition, ease-of-use cognition, and use intention [50]. However, they do not mention how to carry out behavioral intervention strategies in the process of designing the functional structure of remote technology, which may obviously affect patients’ acceptance of remote technology. The eCCM used in this study is a theoretical model of telemedicine intervention proposed by Gee and colleagues [17] based on the CCM. The model includes 8 interdependent and interactive parts: the eCommunity and an informatics framework, health system enhancements, delivery system design enhancements, self-management support enhancements, clinical decision support enhancements, clinical information systems enhancements, addition of eHealth education to the CCM, and communication and addition of the complete feedback loop [17]. A systematic review showed that the implementation of the CCM in primary care could significantly improve the medical effect on patients, improve the quality of life of patients, and reduce the social burden [51]. Evidence showed that eHealth tools could be used to enhance patient self-management behaviors, revise the CCM, and support effective interaction between patients and providers to improve health outcomes [52,53]. Through the components of the eCCM, this study expounded its core characteristics, constructed the functional structure of PeR, and combined the behavioral intervention strategy with the implementation process. The results confirmed the effectiveness of PeR in improving patient dyspnea symptoms, quality of life, and self-efficacy.

The evaluation design of the intervention effect is a necessary part of the health service project design. The multidimensional quality-of-life questionnaire is an efficient evaluation tool in the process of remote intervention. During the course of this study, 82% of patients were tired of using SGRQ and willing to use CAT. Although SGRQ is reliable and effective, it has many items and complex scoring methods, and therefore its widespread use in clinical work is difficult [54,55]. SGRQ is divided into 3 dimensions, with a total of 50 items and a long evaluation time. However, CAT has only 8 questions, with a short evaluation time. CAT is simple and easy to operate, providing a reliable measurement of the COPD health status. CAT and SGRQ have been shown to have a good correlation [56]. In this study, all patients were aged over 60 years. The numerous items of the questionnaire increased the difficulty of implementation. This suggested that patient personal experience of the assessment tool should be taken into account when choosing the online self-report effect assessment tool; a concise and effective assessment tool is the best choice. The combination of convenient, effective, and reliable effect evaluation methods and mobile health is a solution worth considering in telemedicine service.

### Limitations

This study had some limitations. The sample size was small, and the main participants were patients with COPD in China. Hence, data on other races were lacking. Consultation during the implementation of the study may affect the use of PeR, such as the immediate response and extended response time of medical professionals. Despite the best efforts to ensure consistency, the implementation process can still be affected by inevitable factors. All outcomes of the study were self-reported. This is one of the limitations of this study, but it made sense because the study referred to a self-management tool. The clinical outcomes will be addressed in the next study.

### Conclusions

This study used PeR to intervene and manage the pulmonary rehabilitation of patients with COPD at home, which could effectively improve their sense of self-efficacy and quality of life and also alleviate their symptoms. The implementation of PeR confirmed that the eCCM combined with behavioral strategy intervention, based on the self-efficacy theory, could be realized using remote technology. During the application of remote technology, it is worth considering how to construct a free platform that can integrate resources into daily life, especially in the field of COPD and other chronic diseases. Moreover, in the development process of remote technology, we should pay attention to the transformation of theoretical models and combine behavioral intervention at the same time.

## Appendix

Multimedia Appendix 1 CONSORT‐EHEALTH checklist (V 1.6.1).

Multimedia Appendix 2 Computer software copyright registration certificate.

Multimedia Appendix 3 English language version of the functional modules.

## Abbreviations

BMI: body mass index
CAT: chronic obstructive pulmonary disease assessment test
CCM: chronic care model
CONSORT: Consolidated Standards of Reporting Trials
COPD: chronic obstructive pulmonary disease
eCCM: electronic health–enhanced chronic care model
eHealth: electronic health
Ex-SRES: exercise self-regulatory efficacy scale
FtF: face-to-face
FEV1: forced expiratory volume in 1 second
FVC: forced vital capacity
GEE: generalized estimating equation
mMRC: modified Medical Research Council scale
PeR: Pulmonary Internet Explorer Rehabilitation
SGRQ: St George’s Respiratory Questionnaire
SGRQ-A: St George’s Respiratory Questionnaire–Activity
SGRQ-I: St George’s Respiratory Questionnaire–Influence
SGRQ-S: St George’s Respiratory Questionnaire–System
SGRQ-T: St George’s Respiratory Questionnaire–Total
