# Supplementary material for: Evaluating an Intervention Program Using WeChat for Patients With Chronic Obstructive Pulmonary Disease: Randomized Controlled Trial
Source: J Med Internet Res. 2020 Apr 21;22(4):e17089. doi: 10.2196/17089 (PMC7201319; doi:10.2196/17089)
Supplement: Multimedia Appendix 2 [file jmir_v22i4e17089_app2.pdf]

中华人民共和国国家版权局

计算机软件著作权登记证书

证书号：软著登字第4237383号

软 件 名 称：肺e康COPD慢性病管理平台软件 V1.0

著 作 权 人：蒋玉宇;刘凤兰;郭建兰;孙平平

开发完成日期：2019年04月30日

首次发表日期：2019年05月10日

权利取得方式：原始取得

权 利 范 围：全部权利

登 记 号：2019SR0816631

根据《计算机软件保护条例》和《计算机软件著作权登记办法》的规定，经中国版权保护中心审核，对以上事项予以登记。

中华人民共和国国家版权局
计算机软件著作权
登记专用章

No. 04350085

2019年08月06日
