# Supplementary figures and images for: Evaluating an Intervention Program Using WeChat for Patients With Chronic Obstructive Pulmonary Disease: Randomized Controlled Trial
Source: J Med Internet Res. 2020 Apr 21;22(4):e17089. doi: 10.2196/17089 (PMC7201319; doi:10.2196/17089)

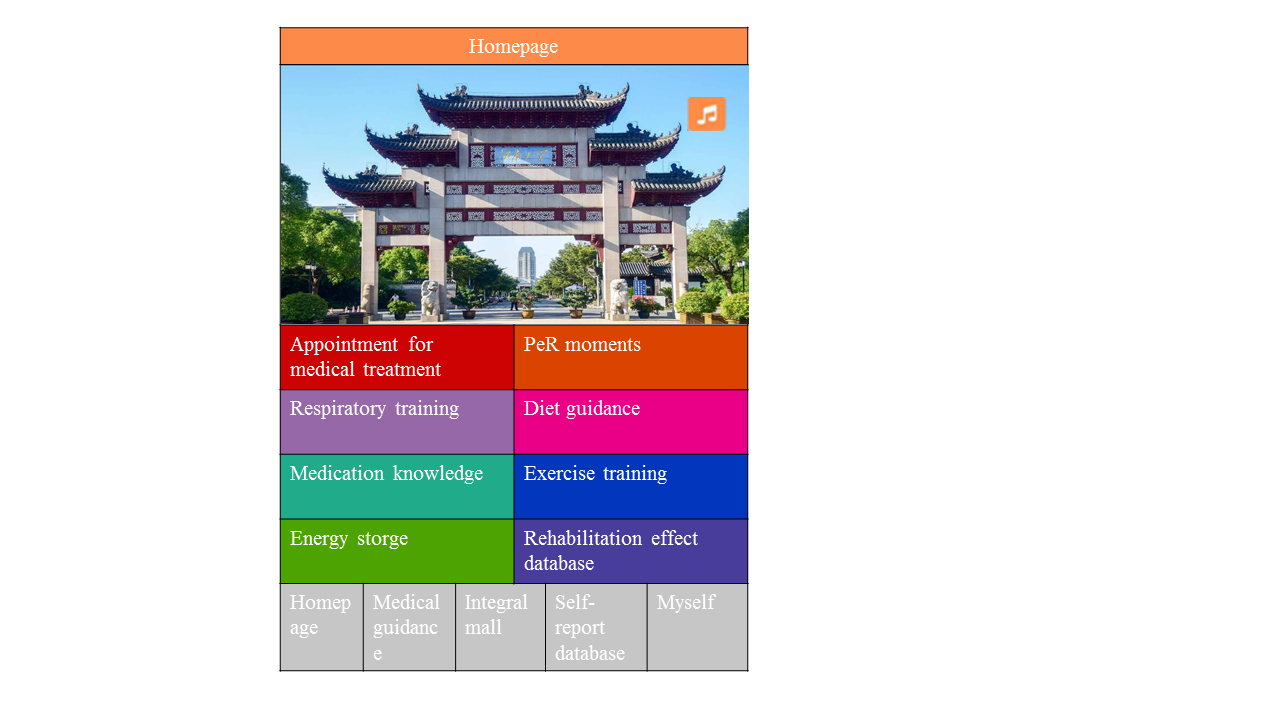

Supplement: Multimedia Appendix 3 [file jmir_v22i4e17089_app3.png]
